# Similarity between structural and proxy estimates of brain connectivity

**DOI:** 10.1177/0271678X231204769

**Published:** 2023-09-29

**Authors:** Aldana Lizarraga, Isabelle Ripp, Arianna Sala, Kuangyu Shi, Marco Düring, Kathrin Koch, Igor Yakushev

**Affiliations:** 1 Department of Nuclear Medicine, 9184 School of Medicine, Klinikum Rechts der Isar, Technical University of Munich, Munich, Germany; 2 Coma Science Group, GIGA Consciousness, University of Liege; Centre du Cerveau2, University Hospital of Liege, Avenue de L'Hôpital 1, Liege, Belgium; 3 Department of Nuclear Medicine, University Hospital Bern, Bern, Switzerland; 4 Medical Image Analysis Center (MIAC AG) and Qbig, Department of Biomedical Engineering, University of Basel, Basel, Switzerland; 5 Department of Neuroradiology, 9184 School of Medicine, Klinikum Rechts der Isar, Technical University of Munich, Munich, Germany

**Keywords:** DWI, fMRI, functional connectivity, molecular connectivity, multimodal imaging

## Abstract

Functional magnetic resonance and diffusion weighted imaging have so far made a major contribution to delineation of the brain connectome at the macroscale. While functional connectivity (FC) was shown to be related to structural connectivity (SC) to a certain degree, their spatial overlap is unknown. Even less clear are relations of SC with estimates of connectivity from inter-subject covariance of regional F18-fluorodeoxyglucose uptake (FDG_cov_) and grey matter volume (GMV_cov_). Here, we asked to what extent SC underlies three proxy estimates of brain connectivity: FC, FDG_cov_ and GMV_cov_. Simultaneous PET/MR acquisitions were performed in 56 healthy middle-aged individuals. Similarity between four networks was assessed using Spearman correlation and convergence ratio (CR), a measure of spatial overlap. Spearman correlation coefficient was 0.27 for SC-FC, 0.40 for SC-FDG_cov_, and 0.15 for SC-GMV_cov_. Mean CRs were 51% for SC-FC, 48% for SC-FDG_cov_, and 37% for SC-GMV_cov_. These results proved to be reproducible and robust against image processing steps. In sum, we found a relevant similarity of SC with FC and FDG_cov_, while GMV_cov_ consistently showed the weakest similarity. These findings indicate that white matter tracts underlie FDG_cov_ to a similar degree as FC, supporting FDG_cov_ as estimate of functional brain connectivity.

## Introduction

Diffusion weighted imaging (DWI) and functional magnetic resonance imaging (fMRI) have so far made a major contribution to delineation of the human brain connectome at the macroscale. Structural connectivity (SC) refers to a physical link between two regions that is inferred from 3D reconstructions of white matter (WM) fiber tracts from DWI data.^1^ In a broad sense, functional connectivity (FC) is defined as ‘statistical dependencies among remote neurophysiological events.^2^ The most common technique to capture FC is fMRI, where neural activity is inferred from variations in blood oxygen level dependent (BOLD) signals over time.^3^ To avoid misunderstandings, we reserve the term “FC” for fMRI-derived connectivity estimates thereafter. A number of studies found that estimates of SC and FC were positively correlated, albeit to a variable degree.^4–9^ Of note, FC was also observed between regions where there was little or no SC,^5,10^ supporting the view that FC is partially mediated by indirect pathways. However, data on spatial overlap between SC and FC at the whole brain level are still missing.

Information on brain connectivity has also been inferred from structural magnetic resonance imaging (sMRI) data, such as T1-weighted images.^11^ In contrast to time series of fMRI, sMRI data are available for analyses as one single image per subject. Thus, sMRI-based connectivity estimation relies on identification of inter-subject covariance patterns at a group level. This approach has produced valuable insights into brain connectivity both in healthy^12,13^ and pathological conditions, such as Alzheimer’s disease ^14^ and schizophrenia.^15^ The covariance patterns have been interpreted as a result of mutual trophic influences mediated by axonal connections or experience-related neural plasticity.^12^ A number of studies found a significant similarity of this connectivity estimate with SC,^6,16^ as well as with FC.^6,17,18^

There is increasing evidence that molecular imaging can effectively contribute to the study of the brain connectome.^19^ Similar to sMRI, PET-based connectivity estimation is commonly performed at a group level. This approach has been successfully applied to PET measures of glucose metabolism,^20–22^ neurotransmission,^23,24^ and pathological protein aggregations.^25–27^ The most popular approach has been PET with 18 F-Fluordesoxyglucose (FDG), sometimes referred to in the literature as metabolic connectivity.^28^ Instead, we now propose the term FDG_cov_, inter-subject covariance of regional FDG-PET measures, to discriminate it from connectivity estimates from functional PET.^29^ FDG_cov_ was found to provide valuable insights into healthy brain function ^30,31^ as well as into pathophysiology and diagnosis of numerous neuropsychiatric disorders.^22,32–36^ So far, only one study has mapped FDG_cov_ to SC.^37^ Specifically, our group found that around a half of FDG_cov_ connections had a structural substrate at the whole brain level. Neither FC nor covariance in gray matter (GM) characteristics were analyzed in our previous study. Furthermore, the cohort included both patients and healthy subjects, and the analysis of similarity was limited to a spatial overlap.^37^

So far, just two studies have investigated the similarity between connectivity estimates derived from at least three imaging techniques.^6,18^ Di and colleagues reported low correlation between FC, covariance in GM volume (GMV_cov_), and FDG_cov_, as well as a limited proportion of overlapping connections. SC was not quantified by the authors. Further, that work included data from multiple sites, acquired with varying imaging protocols and with PET and MRI acquisitions lying up to 4 years apart. Another study compared four estimates of connectivity in human and primate data: FC, covariance in cortical thickness, SC from tract-tracing (in monkeys), and SC from DWI tractography.^6^ They found a poor general agreement, with DWI-SC and FC having the strongest similarity. That study included no PET data.

In the present work we asked to what degree SC may underlie FDG_cov_ in comparison with more established MR-based estimates of brain connectivity, FC and GMV_cov_. Herewith, we explicitly treat SC as reference, because it is indicative of actual anatomical connectivity, reproducible,^38^ and available at a single subject level. In contrast, the estimates FC, FDG_cov_, GMV_cov_ are based on statistical dependencies between regional signals;^2^ we refer to them as *proxy* estimates of brain connectivity thereafter. To address these questions, DWI, fMRI, sMRI, and FDG-PET data were acquired simultaneously in a large group of healthy individuals on a hybrid PET/MR scanner. As both FDG uptake and BOLD signal index neural activity, we hypothesized that SC-FDG_cov_ would be closer to SC-FC rather than to SC-GMV_cov_. This is far from obvious, since unlike FC, FDG_cov_ and GMV_cov_ are quantified from inter-subject variability.^11^

## Materials and methods

### Participants

By means of advertisements in internet and on hospital bulletin boards we recruited healthy, right-handed, German-speaking individuals at the age of 50 to 65 years old. Exclusion criteria were self-reported or objective (test battery) cognitive impairment, history of a neurological or psychiatric disorder, contraindications for MRI, and relevant anomalies on structural MRI images, including cerebrovascular disease. Data of 8 subjects were excluded for the following reasons: excessive motion, i.e., more than 3 mm or 3° maximum displacements during scanning (n = 2), large falx ossifications (n = 1), wrong phase encoding direction in DWI (n = 4), and incomplete data set (n = 1). Thus, the data of 56 individuals (25 females, mean ± SD age: 56 ± 4 years) were available for the present study. The study was performed in accordance with the ethical standards as laid down in the 1964 Declaration of Helsinki. The study was approved by the Federal Office for Radiation Protection and the Ethics Review Board of the University Hospital Klinikum rechts der Isar, Technical University of Munich (project number 399/13). All participants provided written, informed consent.

### Image acquisition

The participants were scanned in a 3T PET/MR Siemens Biograph mMR scanner with a vendor-supplied 16-channel head coil. They were instructed to fast for six hours prior to the scan session. After the intravenous injection of on average 102 ± 5 (SD) MBq of 18 F-FDG, the participants stayed comfortably in a quiet, dimly lit room, with closed eyes. Reconstructed PET images (30–60 min post-injection) had a voxel size of 1.04 × 1.04 × 2.03 mm^3^. Anatomical T1-weighted images were acquired with a magnetization‐prepared rapid gradient‐echo (MP‐RAGE) sequence with a voxel size of 1.0 × 1.0 × 1.0 mm^3^. DWI was performed using a single-shot EPI sequence of voxel size 2 × 2 × 2 mm^3^, with 30 diffusion directions with b = 800 s/mm^2^ and one volume with b = 0 s/mm^2^. For the fMRI acquisition, which lasted 8 min, participants were instructed to stay awake, close their eyes and think of nothing in particular. 212 volumes were acquired using a Prospective Acquisition Correction EPI sequence with voxel size of 3.0 × 3.0 × 3.0 mm^3^. An ultra-short echo time sequence was acquired for attenuation correction of the PET data. A dual echo gradient echo sequence was acquired to correct the DWI images for susceptibility induced distortions. Details of the PET and MR acquisitions are given in the supplementary material to our recent article.^39^

### Grey matter parcellation

Estimation of connectivity indices from four imaging techniques required us to make a number of methodological decisions. One of the major challenges was the choice of a parcellation scheme from a vast variety of available brain atlases. Finally, we decided to use the Automated Anatomical Labeling 2 (AAL2) atlas^40^ for the following reasons: a) it includes cortex, cerebellum, and subcortical regions, b) it has a lower proportion of small regions, an important advantage in light of potential partial volume effects in PET data, c) it is more robust than other atlases against non-linear registrations (data not shown), d) it has a simple nomenclature, e) it is the most widely used atlas, making a comparison with the literature more straightforward. Since AAL2 regions are defined in a way that their borders extend beyond GM, we sampled only GM part of those regions (see below). Another important methodological issue was a registration approach. We decided to keep the images in the native (individual) space and transform the atlas from the MNI space to the individual space, in order to reduce data manipulation and have a better visual control over accuracy of registration. Thus, the AAL2 parcellation was non-linearly transformed from the MNI152 T1 space to the individual T1 space using Advanced Normalization Tools (ANTs).^41^ Afterwards, T1 images were segmented using SPM12 (https://www.fil.ion.ucl.ac.uk/spm/software/spm12/). GM probability maps were then binarized at a probability of 0.5, followed by parcellation. Certain regions of the original parcellation were combined or removed in order to minimize the number of small regions (volume <512 mm^3^, i.e. 2 × FWHM in all directions). Finally, we obtained a parcellation of 106 regions. The list of regions and further details are given in the Supplementary Material.

### Structural connectivity network

We obtained SC networks from DWI data using tools of the FMRIB Software Library (FSL). After visual inspection of the images by means of FSLeyes, we estimated the field map with *fsl_prepare_fieldmap* and corrected for susceptibility induced distortions, eddy currents, inter-volume movement, and signal dropout using *eddy*.^42^ Then, we extracted brain tissue with BET^43^ and fit the Ball and Sticks diffusion model with N = 2 using BedpostX.^44^ Afterwards, we performed whole brain probabilistic tractography using a WM seeding approach with ProbtrackX. To this end, 0.5 thresholded masks of GM, WM, and CSF were used as target, seed and exclusion masks, respectively. To allow transformation of the masks from the T1 space to the DWI space, in which the tractography was performed, a linear transformation was provided to ProbtrackX. As an estimate of SC we used the number of streamlines connecting two GM regions and normalized them by the surface area of those regions in the WM-GM interface to compensate for surface-driven effects on streamline counts.^45,46^ In order to construct a group SC network, we resampled the connection weights, i.e. strength, of each subject to Gaussian distribution^5,47^ and calculated an average across subjects. Following multiple studies, we applied a proportional threshold to retain connections present in more than 75% of subjects.^48–50^ This resulted in a network density of 34%.

### Functional connectivity network

FC was inferred from fMRI data. The diagnostic tool *tsdiffana* was utilized in MATLAB v2020a software (The MathWorks Inc., Natick, Massachusetts, USA) to detect corrupted frames that might not be noticed visually. After removal of the first three volumes of the BOLD series, the images were corrected for slice timing and realigned to the volume acquired temporally in the middle using SPM12. We created WM and CSF masks applying a threshold of 0.99 to the probability maps from the T1 segmentation. Those masks, as well as the GM parcellation, were linearly transformed from the T1 space to the BOLD space using the FSL registration tool FLIRT with a nearest neighbor interpolation.^51^ After that, we regressed the WM and CSF signal components using *fsl_glm.* Motion was not regressed out, as this may reduce reliability.^52^ Instead, we checked that none of the included subjects showed an excessive motion (>3 mm or >3°). A temporal band-pass filter (0.009–0.080 Hz) was applied to reduce non-BOLD high frequency signals due to heart rate and breathing and lower frequency signals due to scanner instability.^4^ Next, we extracted the signal from each region and calculated the Pearson correlation between each pair of regions. In order to build a group FC network, we applied a Fisher z-transformation to the correlation coefficients, averaged them across subjects and transformed them back to Pearson correlation coefficients.^18^

### Covariance networks

A group network of FDG_cov_ was estimated from FDG-PET images. After a linear registration of PET images to the individual T1 space with SPM, FDG uptake was extracted from each region and normalized by mean GM uptake.^53^ Finally, Pearson correlations were calculated between each pair of regions across subjects.

To keep the cerebellum and subcortical structures in the analyses, we decided to use GM volume rather than GM thickness as morphological feature. GM volume (GMV) was calculated for each region as a sum of GM probabilities multiplied by the voxel size. Afterwards, we regressed out the total GMV of each subject.^54^ Finally, Pearson correlation of the residuals between pairs of regions was calculated across subjects to obtain a symmetric 106x106 matrix.

### Similarity between the networks

To assess similarity between the networks in terms of strength, we calculated Spearman correlations between connection weights of a network pair. This non-parametric test was chosen, as the connection weights were not normally distributed (Kolmogorov-Smirnov test). As the biological meaning of negative FC as well as negative FDG_cov_ and GMV_cov_ is still under discussion,^55^ we in addition re-calculated the correlations for positive connections weights only.^18,56^

To assess similarity between the networks in terms of spatial distribution, we calculated convergence ratio (CR), the number of common connections divided by the average of the connections in a network pair. This index is equivalent to Dice similarity coefficient. To this end, the networks need to be binarized via thresholding. Common approaches are 1) removal of connections below a minimum connection weight,^57^ 2) removal of connections below a desired density/sparsity,^58^ where the sparsity is defined as the number of null elements in its matrix divided by the total number of elements,^50^ and 3) removal of connections above certain *p*-values of correlation coefficients.^23,31^ While the approaches 1) and 3) produce networks with a different number of connections, 2) produces networks with the same number of connections but a different minimum weight. As the spatial overlap between binary networks is affected by their sparsity, but independent of the connection weight, we decided to use the approach 2). Thus, CR between all network pairs was calculated at a sparsity range of 65.8% to 80%. The lower limit was imposed by the sparsity of the group SC network, which was 65.8% after the proportional thresholding step. The upper limit ensures that all networks are connected.^16^ In graph theory, a network is connected, if one can go from a region A to a region B by following a path (link succession). Further, we computed CR expected by chance as described in the Supplementary Material.

As secondary analyses, we calculated similarity between the proxy estimates of brain connectivity only. Herewith, all possible connections, i.e., irrespective of SC, were considered. We note, however, that this comparison should be treated *with caution*, as in the absence of a reference standard, it is not clear which connections are true and which are artifactual.

### Robustness analyses

To assess robustness of our results, we repeated the analyses under different conditions, namely:

- Test-retest reproducibility, i.e., we analyzed imaging data acquired under the same conditions approximately 8 weeks later.^39^
- Omitting Gaussian resampling of SC, i.e., each element of the group SC matrix was calculated as average of normalized streamline counts across subjects.
- Omitting SC thresholding, i.e., no threshold was applied to the group SC matrix.
- Applying a more liberal proportional threshold of 50% to the SC matrix.
- Regressing out a distance between the regions. Since brain networks are spatially embedded, distance between regions might be a confound when estimating similarity between networks.^7^ To take that into account, we calculated Euclidean distance between ROI centroids in the native space of each subject, followed by a linear regression of mean distances across subjects from the weight distribution of SC and proxy estimates of brain connectivity. Resultant residuals were used to recalculate CRs. In addition, partial Spearman correlations between connection weights were calculated using distance as covariate.
- Limiting the analysis to the cerebral cortex. To this end, vermis, cerebellum, and subcortical structures were excluded from the analysis, resulting in a parcellation of 80 cortical regions.

## Results

**Figure 1.**
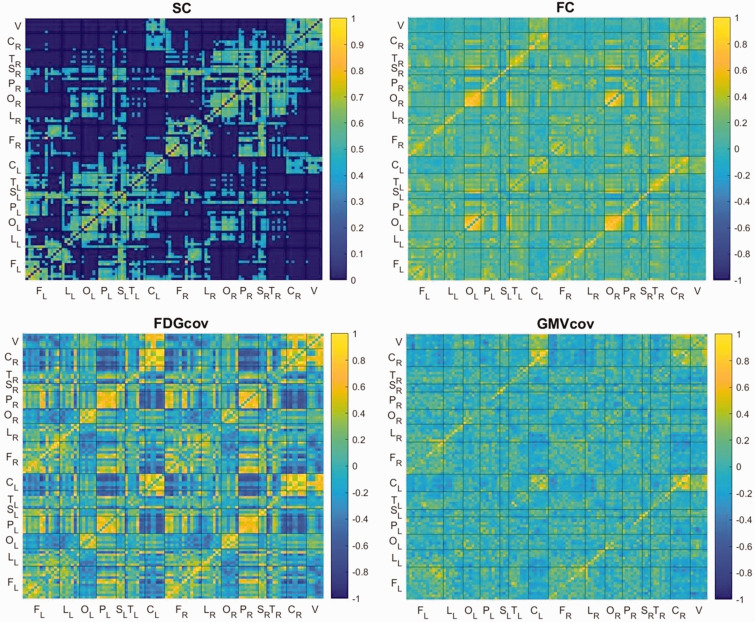
Group matrix for each connectivity estimate. The color bars indicate connection weights. The 106 regions were grouped into the following structures: frontal (F), limbic (L), occipital (O), parietal (P), subcortical (S), temporal (T), cerebellar hemispheres (C), and vermis (V). The subscript indicates the left (L) and right (R) hemisphere.

Figure 1 presents the adjacency matrices for each brain connectivity estimate, where each entry indicates the connection weight, i.e., strength of a link between regions. The SC matrix had a sparsity of 65.8%; no region was disconnected (Figure 1a). Unthresholded SC matrix is shown in Supplementary Figure 1.

**Figure 2.**
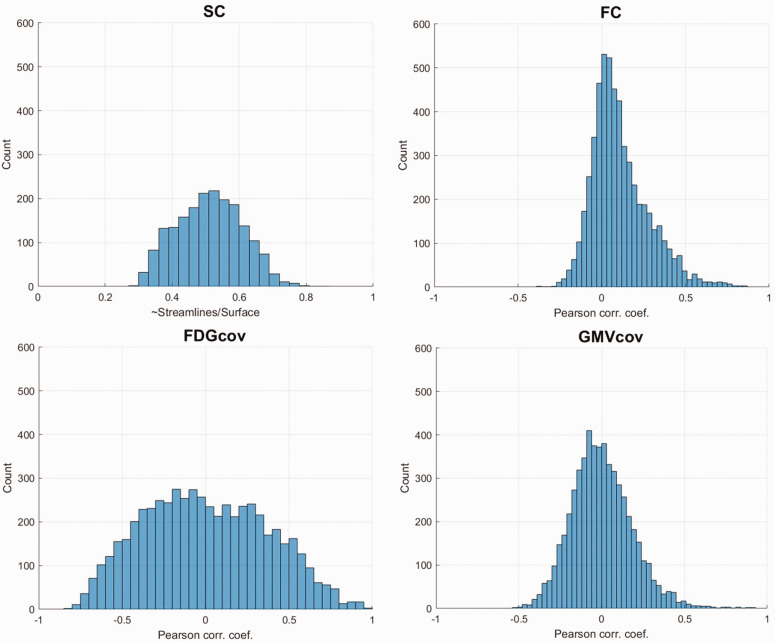
Distribution of edge/connection weights for each estimate.

Figure 2 shows histograms of connection weights. The connection weight distributions for all networks was unimodal, with a percentage of negative weights equal to 26% for FC, 52% for FDG_cov_, and 53% for GMV_cov_. Histograms of connection weights corresponding to the unthresholded SC network are shown in Supplementary Figure 2.

**Figure 3.**
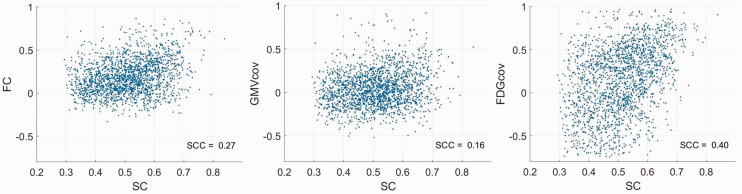
Scatter plots of connection weights and Spearman correlation coefficients for SC and the proxy estimates.

The scatterplots and Spearman correlation coefficients (SCC) between SC and the proxy estimates are shown in Figure 3. All SCC were positive and statistically significant (*p*’s < 0.001, Bonferroni corrected), decreasing in the order *r = *0.40 for SC-FDG_cov_, *r = *0.27 for SC-FC, and *r = *0.16 for SC-GMV_cov_. The correlations for positive weights only are shown in Supplementary Figure 3. The coefficients decreased in the order *r = *0.31 for SC-FC, *r = *0.26 for SC-FDG_cov_, and *r = *0.18 for SC-GMV_cov_ (*p*’s < 0.001, Bonferroni corrected).

**Figure 4.**
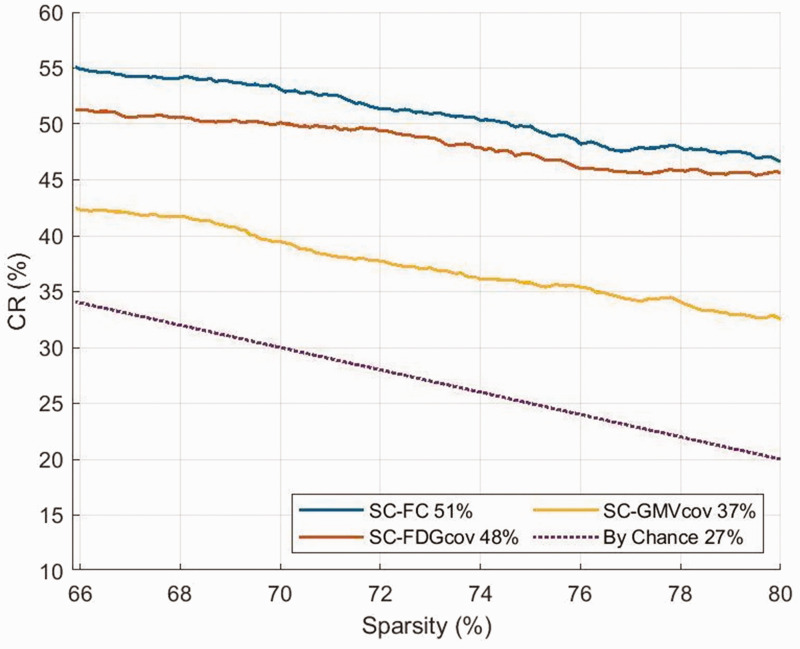
Convergence ratio (CR) between SC and proxy estimates as a function of sparsity level. The percentages indicate mean CRs over the sparsity range.

Spatial overlap as quantified by CR was substantially higher than that expected by chance for all networks (Figure 4). Mean CRs were 51% for SC-FC, 48% for SC-FDG_cov_, and 37% for SC-GMV_cov_.

**Figure 5.**
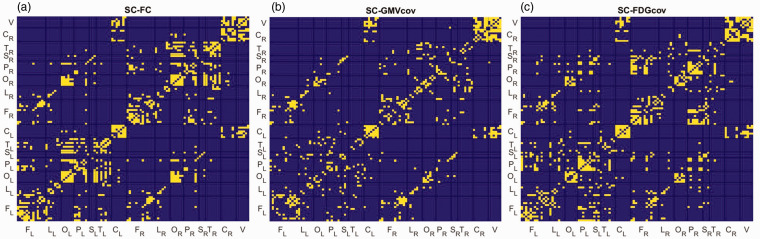
Overlap matrices between SC and proxy estimates as a function of sparsity level. Yellow elements indicate common connections after thresholding the networks at 80% sparsity.

Figure 5 presents an overlap between SC and the proxy estimates at 80% sparsity. Overall, it was stronger for intralobe connections and homotopic interhemispheric connections, especially in the cerebellum (hemispheres and vermis). In addition, FDG_cov_ and FC stronger overlapped with SC in the occipital and frontal lobes.

SCC between weights of the proxy estimates were *r = *0.30 for FC-FDG_cov_, *r = *0.25 for FC-GMV_cov_, and *r = *0.22 for FDG_cov_-GMV_cov_ (Supplementary Figure 4). CRs for the proxy estimates were 46% for FDG_cov_-FC, 43% for GMV_cov_-FC, and 40% for FDG_cov_-GMV_cov_ (Supplementary Figure 5).

Results of the robustness analyses are presented in Table 1. Overall, the measures of similarity did not change substantially. First, the entire analysis proved to be highly reproducible, including the limits of the sparsity interval (66.1–79.8%) (a). Omitting Gaussian resampling affected neither sparsity of the SC matrix nor the ranking of similarities between the networks (b). Omitting SC thresholding leaded to a denser SC matrix with a sparsity of 13.3% (c). A resultant wider sparsity interval did not change significantly SCC, but increased systematically CRs, making them closer to CR by chance (c). Application of a more liberal proportional threshold of 50% to the SC matrix produced sparsity of 55%. Beside an expected increase in CRs, the similarity metrics showed the same behavior as before (d). Regressing out the distance between regions altered maximum sparsity, at which all networks are to be connected, from 80% to 64.7%. This precluded us from applying the connectedness criterion to set the upper limit of the sparsity range. Therefore, we kept the previous sparsity rage (65.8–80.0%). As expected, both SCCs and CRs decreased when distance between regions was regressed out, but CR values were still well above those expected by chance (e). Finally, when the analyses were limited to the cortex (f), SCC and CR values remained similar. The measures of similarity between the proxy estimates only did not change substantially, too (Supplementary Table 2).

**Table 1.** Robustness analyses.

	SCC						
	O	a)	b)	c)	d)	e)	f)
SC-FDG_cov_	0.40	0.41	0.42	0.32	0.38	0.26	0.38
SC-FC	0.27	0.24	0.27	0.31	0.29	0.11	0.34
SC-GMV_cov_	0.16	0.16	0.17	0.17	0.15	0.01	0.17
	CR						
SC-FDG_cov_	48	48	49	63	50	37	49
SC-FC	51	50	51	66	54	41	51
SC-GMV_cov_	37	37	37	59	42	29	36
CR by chance	27	27	27	53	32	27	30
Min sparsity	66	66	66	13	55	66	64
Max sparsity	80	80	80	80	80	80	77

Similarity metrics obtained under modified conditions relative to the original (O) ones: a) Test-retest reproducibility, b) Omitting the Gaussian resampling step of SC, c) Omitting the proportional threshold to SC matrix, d) Applying a 50% proportional threshold to the SC matrix, e) Regressing out distance from all connectivity estimates, f) limiting the analysis to the cerebral cortex. SCC: Spearman correlation coefficient; CR: mean convergence ratio computed over the corresponding sparsity range [min sparsity, max sparsity].

## Discussion

In the present study, we examined similarity between SC and proxy estimates of brain connectivity FC, GMV_cov_, and FDG_cov_. All SCCs as index of similarity strength were significant, with a low to moderate degree. CRs as index of spatial similarity appeared to be higher than that by chance for all pairs of the estimates. The strongest similarity with SC was found for FC and FDG_cov_, while GMV_cov_ consistently showed the weakest similarity. Of note, comprehensive additional analyses confirmed robustness of the findings.

Anatomical connections between brain regions are considered as structural core of brain connectivity.^59^ To what degree does this core explain patterns of functional connectivity? By functional connectivity we explicitly mean here estimates of brain connectivity from measures of neural activity, such as fMRI and FDG*-*PET. The present results indicate that around a half of functional connections, both from fMRI and FDG-PET, are underlied by SC. While *spatial* similarity in terms of CR was slightly higher for fMRI, similarity in terms of SCC was higher for FDG_cov_. This is not implausible, because not identical connections were considered in the analyses. To calculate CR, 34% of the strongest connections in each network were considered. Hence, negative connections were excluded. In contrast, the Spearman correlations were restricted to structurally consistent connections, independently of their strength and sign. Thus, the relevance of a structural connection in terms of strength is more similar to that of FDG_cov_ than FC, while the strongest FC spatially overlap somewhat better with structural connections than the strongest FDG_cov_ connections. As expected, limiting the correlation analyses to positive connection weights produced the same order of similarity as CR, i.e. SC-FC > SC-FDG_cov_.

While FDG-PET has been increasingly utilized in the field of brain connectivity,^19^ the meaning of FDG_cov_ and its relationship with the more established MRI-based estimates of brain connectivity has been unclear. The present study indicates that SC as estimated with DWI underlie FDG_cov_ and FC to a similar degree. A relevant relationship between FC and FDG_cov_ is further supported by our additional analyses of similarity between proxy estimates of connectivity, where FC and FDG_cov_ showed the highest correlation and spatial overlap with each other. This result is intriguing, given important differences between the estimates, especially in respect to the targeted process, acquisition mode, and modeling approach.^60^ We speculate that similarity might be even higher when indices of brain connectivity are derived from temporal correlations in dynamic FDG-PET data.^29^ In contrast, similarity between GMV_cov_ and FDG_cov_ was lowest, even somewhat lower than that between GMV_cov_ and FC. These observations strongly support FDG_cov_ as index of functional connectivity rather than a sort of statistical artifact of inter-subject correlations.^29,61^

The first attempt to compare SC and FDG_cov_ networks has been undertaken by our group very recently.^37^ We found a spatial overlap of 55%, i.e. very similar value to that of the present work (48%). These similar results are rather surprising given a number of important differences between the studies in respect to study design and methods, e.g., population (patients and healthy subjects vs. healthy subjects only), atlases, number of regions (62 vs. 106), modeling approach of FDG_cov_ (sparse inverse covariance estimation vs. correlation), method of tractography (deterministic vs. probabilistic), and index of SC (fractional anisotropy vs. normalized streamline counts). Despite the methodological differences the present work supports our previous conclusion that around half of FDG_cov_ connections may have a structural substrate at the whole brain level.

Similarity between SC and FC has been extensively addressed in the literature, both in healthy and pathological conditions. Although the studies coincide in a positive association between these two estimates, the reported correlation values vary from weak to good,^4–7,47,62^ likely due to methodological differences between the studies. The correlation coefficients in the present study are well within this range. We are not aware of any data on spatial overlap between SC and FC at the whole brain level. Here, we found a CR of 51%. In other words, roughly a half of functional connections have a structural substrate.

We explored for the first time GM covariance networks in combination with FDG_cov_, SC, and FC in the same healthy subjects. So far, the biological mechanisms behind regional GM covariance have not been well understood. The most widespread hypotheses point at mutually trophic factors mediated by axonal connections,^63^ activity-dependent processes,^64^ common experience-related plasticity,^63^ and genetics.^65^ In the present study, GMV_cov_ showed the weakest similarity with the other estimates. Of note, our quantitative results closely resemble previously reported ones, despite a number of methodological differences. In particular, Gong et al. compared patterns of covariance of GM thickness with SC across the entire cerebral cortex.^16^ For a similar sparsity level, they reported a CR of 33 to 37%, while we found a CR of 33 to 36%. Our results are furthermore well in line with those of Reid et al., who reported the weakest similarity for covariance of GM thickness with SC and FC.^6^ Their SCCs were, however, higher, likely due to a better data quality and a lower resolution parcellation.^5,6^ Further, Di and colleagues compared GMV_cov_, FC, and FDG_cov_ networks through Spearman correlations and percentage of overlap.^18^ Their SCCs were very close to ours and, alike, those for GMV_cov_ were the weakest. Like in the present study, similarity between the proxy estimates decreased in the following order FDG_cov_-FC > FDG_cov_-GMV_cov_ > FC-GMV_cov_ at the majority of sparsity levels. These observations support the conclusion above that FDG_cov_ as estimate of functional brain connectivity is closer to FC than to GMV_cov_.

In this study, we decided to omit any streamline-length correction of SC weights. It is known that long-distance connections detected through tractography are fewer and weaker than short-distance connections.^66^ To adjust for this, FSL’s length correction multiplies the connectivity distribution by the expected length of pathways. However, this effect has also been observed in invasive tract tracing studies, such that the WM seeding approach applied here was suggested to mitigate such a bias.^67^ Nevertheless, we took into account the distance between regions in our robustness analyses. A systematic decrease in the similarity metrics suggests that spatial embedding might contribute to network similarity.^4,5,7^

A limitation of this study is a relatively low b-value as well as a low number of diffusion directions. Yet, while a higher b-value would increase the diffusion weighting of the images, it might also reduce their signal-to-noise ratio. Along these lines, increasing the number of directions would require a longer acquisition time of the DWI sequence, limiting practicability of multimodal imaging protocols as ours. Further, a required number of diffusion directions as well as an optimal b-value depend on the diffusion model to be applied.^68^ Of note, the FSL *ball and sticks* model, as applied here, was shown to robustly detect crossing fibers in DWI data acquired with parameters close to ours.^46^

In summary, we found a relevant similarity of SC with FC and FDG_cov_, while GMV_cov_ consistently showed the weakest similarity. These results indicate that SC underlies FDG_cov_, inter-subject covariance of FDG uptake, to a similar degree as group FC, an established estimate of brain connectivity. This work underpins FDG_cov_ as index of *functional* brain connectivity. Future studies should compare properties of the different connectomes using e.g., graph theory metrics. Further, dependence of FDG_cov_ on intensity normalization should be explored.

## Supplemental Material

sj-pdf-1-jcb-10.1177_0271678X231204769 - Supplemental material for Similarity between structural and proxy estimates of brain connectivity Supplemental material, sj-pdf-1-jcb-10.1177_0271678X231204769 for Similarity between structural and proxy estimates of brain connectivity by Aldana Lizarraga, Isabelle Ripp, Arianna Sala, Kuangyu Shi, Marco Düring, Kathrin Koch and Igor Yakushev in Journal of Cerebral Blood Flow & Metabolism

## Data Availability

Data and codes are available upon request.

## References

[1] Smith R Raffelt D Tournier JD , et al. Quantitative streamlines tractography: methods and inter-subject normalisation. Open Science Framework 2020, https://osf.io/c67kn (accessed 9 March 2023).

[2] Friston KJ. Functional and effective connectivity: a review. Brain Connect 2011; 1: 13–36. 22432952 10.1089/brain.2011.0008

[3] Lowe MJ Mock BJ Sorenson JA. Functional connectivity in single and multislice echoplanar imaging using resting-state fluctuations. NeuroImage 1998; 7: 119–132. 9558644 10.1006/nimg.1997.0315

[4] Skudlarski P Jagannathan K Calhoun VD , et al. Measuring brain connectivity: diffusion tensor imaging validates resting state temporal correlations. NeuroImage 2008; 43: 554–561. 18771736 10.1016/j.neuroimage.2008.07.063 PMC4361080

[bibr5-0271678X231204769] Honey CJ Sporns O Cammoun L , et al. Predicting human resting-state functional connectivity from structural connectivity. Proc Natl Acad Sci U S A 2009; 106: 2035–2040. 19188601 10.1073/pnas.0811168106 PMC2634800

[bibr6-0271678X231204769] Reid AT Lewis J Bezgin G , et al. A cross-modal, cross-species comparison of connectivity measures in the primate brain. NeuroImage 2016; 125: 311–331. 26515902 10.1016/j.neuroimage.2015.10.057

[bibr7-0271678X231204769] Garcés P Pereda E Hernández‐Tamames JA , et al. Multimodal description of whole brain connectivity: a comparison of resting state MEG, fMRI, and DWI. Hum Brain Mapp 2016; 37: 20–34. 26503502 10.1002/hbm.22995 PMC5132061

[bibr8-0271678X231204769] Zimmermann J Griffiths J Schirner M , et al. Subject specificity of the correlation between large-scale structural and functional connectivity. Netw Neurosci 2019; 3: 90–106. 30793075 10.1162/netn_a_00055 PMC6326745

[9] Messé A. Parcellation influence on the connectivity‐based structure–function relationship in the human brain. Hum Brain Mapp 2020; 41: 1167–1180. 31746083 10.1002/hbm.24866 PMC7267927

[10] Koch MA Norris DG Hund-Georgiadis M. An Investigation of functional and anatomical connectivity using magnetic resonance imaging. NeuroImage 2002; 16: 241–250. 11969331 10.1006/nimg.2001.1052

[11] Seghier ML Price CJ. Interpreting and utilising intersubject variability in brain function. Trends Cogn Sci 2018; 22: 517–530. 29609894 10.1016/j.tics.2018.03.003 PMC5962820

[12] Romero-Garcia R Whitaker KJ Váša F , NSPN Consortium et al. Structural covariance networks are coupled to expression of genes enriched in supragranular layers of the human cortex. NeuroImage 2018; 171: 256–267. 29274746 10.1016/j.neuroimage.2017.12.060 PMC5883331

[13] Seidlitz J Váša F Shinn M , NSPN Consortium et al. Morphometric similarity networks detect microscale cortical organization and predict Inter-Individual cognitive variation. Neuron 2018; 97: 231–247.e7. Jan 29276055 10.1016/j.neuron.2017.11.039 PMC5763517

[14] Montembeault M Rouleau I Provost JS , Alzheimer's Disease Neuroimaging Initiative et al. Altered gray matter structural covariance networks in early stages of alzheimer’s disease. Cereb Cortex 2016; 26: 2650–2662. 25994962 10.1093/cercor/bhv105 PMC4869809

[15] Palaniyappan L Hodgson O Balain V , et al. Structural covariance and cortical reorganisation in schizophrenia: a MRI-based morphometric study. Psychol Med 2019; 49: 412–420. 29729682 10.1017/S0033291718001010

[16] Gong G He Y Chen ZJ , et al. Convergence and divergence of thickness correlations with diffusion connections across the human cerebral cortex. NeuroImage 2012; 59: 1239–1248. 21884805 10.1016/j.neuroimage.2011.08.017

[17] Reid AT Hoffstaedter F Gong G , et al. A seed-based cross-modal comparison of brain connectivity measures. Brain Struct Funct 2017; 222: 1131–1151. 27372336 10.1007/s00429-016-1264-3 PMC5205581

[18] Di X Gohel S Thielcke A , et al. Do all roads lead to Rome? A comparison of brain networks derived from inter-subject volumetric and metabolic covariance and moment-to-moment hemodynamic correlations in old individuals. Brain Struct Funct 2017; 222: 3833–3845. 28474183 10.1007/s00429-017-1438-7 PMC10650976

[19] Sala A Lizarraga A Caminiti SP , et al. Brain connectomics: time for a molecular imaging perspective? Trends Cogn Sci 2023; 27: 353–366. 36621368 10.1016/j.tics.2022.11.015 PMC10432882

[20] Moeller JR Strother SC Sidtis JJ , et al. Scaled subprofile model: a statistical approach to the analysis of functional patterns in positron emission tomographic data. J Cereb Blood Flow Metab 1987; 7: 649–658. 3498733 10.1038/jcbfm.1987.118

[bibr21-0271678X231204769] Sala A Caminiti SP Iaccarino L , et al. Vulnerability of multiple large‐scale brain networks in dementia with Lewy bodies. Hum Brain Mapp 2019; 40: 4537–4550. 31322307 10.1002/hbm.24719 PMC6917031

[22] Caminiti SP Tettamanti M Sala A , et al. Metabolic connectomics targeting brain pathology in dementia with Lewy bodies. J Cereb Blood Flow Metab 2017; 37: 1311–1325. 27306756 10.1177/0271678X16654497 PMC5453453

[23] Veronese M Moro L Arcolin M , et al. Covariance statistics and network analysis of brain PET imaging studies. Sci Rep 2019; 9: 2496. 30792460 10.1038/s41598-019-39005-8 PMC6385265

[24] Verger A Horowitz T Chawki MB , et al. From metabolic connectivity to molecular connectivity: application to dopaminergic pathways. Eur J Nucl Med Mol Imaging 2020; 47: 413–424. 31741020 10.1007/s00259-019-04574-3

[25] Hoenig MC Bischof GN Seemiller J , et al. Networks of tau distribution in Alzheimer’s disease. Brain 2018; 141: 568–581. 29315361 10.1093/brain/awx353

[bibr26-0271678X231204769] Ossenkoppele R Iaccarino L Schonhaut DR , et al. Tau covariance patterns in Alzheimer’s disease patients match intrinsic connectivity networks in the healthy brain. Neuroimage Clin 2019; 23: 101848. 31077982 10.1016/j.nicl.2019.101848 PMC6510968

[27] Pereira JB Ossenkoppele R Palmqvist S , et al. Amyloid and tau accumulate across distinct spatial networks and are differentially associated with brain connectivity. eLife 2019; 8: e50830. 31815669 10.7554/eLife.50830 PMC6938400

[28] Yakushev I Drzezga A Habeck C. Metabolic connectivity: methods and applications. Curr Opin Neurol 2017; 30: 677–685. 28914733 10.1097/WCO.0000000000000494

[29] Jamadar SD Ward PGD Liang EX , et al. Metabolic and hemodynamic resting-state connectivity of the human brain: a high-temporal resolution simultaneous BOLD-fMRI and FDG-fPET multimodality study. Cereb Cortex 2021; 31: 2855–2867. 33529320 10.1093/cercor/bhaa393

[30] Zou N Chetelat G Baydogan MG , et al. Metabolic connectivity as index of verbal working memory. J Cereb Blood Flow Metab 2015; 35: 1122–1126. 25785830 10.1038/jcbfm.2015.40 PMC4640275

[31] Huang Q Zhang J Zhang T , et al. Age-associated reorganization of metabolic brain connectivity in Chinese children. Eur J Nucl Med Mol Imaging 2020; 47: 235–246. 31520171 10.1007/s00259-019-04508-z

[32] Perani D Farsad M Ballarini T , et al. The impact of bilingualism on brain reserve and metabolic connectivity in Alzheimer’s dementia. Proc Natl Acad Sci U S A 2017; 114: 1690–1695. 28137833 10.1073/pnas.1610909114 PMC5320960

[bibr33-0271678X231204769] Titov D Diehl-Schmid J Shi K , et al. Metabolic connectivity for differential diagnosis of dementing disorders. J Cereb Blood Flow Metab 2017; 37: 252–262. 26721391 10.1177/0271678X15622465 PMC5363743

[bibr34-0271678X231204769] Pagani M Giuliani A Öberg J , et al. Progressive disintegration of brain networking from normal aging to Alzheimer disease: analysis of independent components of ^18^F-FDG PET data. J Nucl Med 2017; 58: 1132–1139. 28280223 10.2967/jnumed.116.184309

[bibr35-0271678X231204769] Verger A Roman S Chaudat RM , et al. Changes of metabolism and functional connectivity in late-onset deafness: evidence from cerebral 18F-FDG-PET. Hear Res 2017; 353: 8–16. 28759745 10.1016/j.heares.2017.07.011

[36] Shim HK Lee HJ Kim SE , et al. Alterations in the metabolic networks of temporal lobe epilepsy patients: a graph theoretical analysis using FDG-PET. Neuroimage Clin 2020; 27: 102349. 32702626 10.1016/j.nicl.2020.102349 PMC7374556

[37] Yakushev I Ripp I Wang M , et al. Mapping covariance in brain FDG uptake to structural connectivity. Eur J Nucl Med Mol Imaging 2022; 49: 1288–1297. 34677627 10.1007/s00259-021-05590-y PMC8921091

[38] Messaritaki E Dimitriadis SI Jones DK. Optimization of graph construction can significantly increase the power of structural brain network studies. NeuroImage 2019; 199: 495–511. 31176831 10.1016/j.neuroimage.2019.05.052 PMC6693529

[39] Ripp I Emch M Wu Q , et al. Adaptive working memory training does not produce transfer effects in cognition and neuroimaging. Transl Psychiatry 2022; 12: 512. 36513642 10.1038/s41398-022-02272-7 PMC9747798

[40] Rolls ET Joliot M Tzourio-Mazoyer N. Implementation of a new parcellation of the orbitofrontal cortex in the automated anatomical labeling atlas. NeuroImage 2015; 122: 1–5. 26241684 10.1016/j.neuroimage.2015.07.075

[41] Avants BB Tustison NJ Song G , et al. A reproducible evaluation of ANTs similarity metric performance in brain image registration. NeuroImage 2011; 54: 2033–2044. 20851191 10.1016/j.neuroimage.2010.09.025 PMC3065962

[42] Andersson JLR Sotiropoulos SN. An integrated approach to correction for off-resonance effects and subject movement in diffusion MR imaging. NeuroImage 2016; 125: 1063–1078. 26481672 10.1016/j.neuroimage.2015.10.019 PMC4692656

[43] Smith SM. Fast robust automated brain extraction. Hum Brain Mapp 2002; 17: 143–155. 12391568 10.1002/hbm.10062 PMC6871816

[44] Behrens TEJ Berg HJ Jbabdi S , et al. Probabilistic diffusion tractography with multiple fibre orientations: what can we gain? NeuroImage 2007; 34: 144–155. 17070705 10.1016/j.neuroimage.2006.09.018 PMC7116582

[45] van den Heuvel MP Sporns O. Rich-club organization of the human connectome. J Neurosci 2011; 31: 15775–15786. 22049421 10.1523/JNEUROSCI.3539-11.2011 PMC6623027

[46] Bonilha L Gleichgerrcht E Fridriksson J , et al. Reproducibility of the structural brain connectome derived from diffusion tensor imaging (Hayasaka S, ed.). PLoS ONE 2015; 10: e0135247. 26332788 10.1371/journal.pone.0135247 PMC4557836

[47] Hagmann P Sporns O Madan N , et al. White matter maturation reshapes structural connectivity in the late developing human brain. Proc Natl Acad Sci U S A 2010; 107: 19067–19072. 20956328 10.1073/pnas.1009073107 PMC2973853

[48] Barbagallo G Caligiuri ME Arabia G , et al. Structural connectivity differences in motor network between tremor-dominant and nontremor Parkinson’s disease: structural network in PD phenotypes. Hum Brain Mapp 2017; 38: 4716–4729. 28631404 10.1002/hbm.23697 PMC6866900

[bibr49-0271678X231204769] Nomi JS Schettini E Broce I , et al. Structural connections of functionally defined human insular subdivisions. Cereb Cortex 2018; 28: 3445–3456. 28968768 10.1093/cercor/bhx211 PMC6132280

[50] Buchanan CR Bastin ME Ritchie SJ , et al. The effect of network thresholding and weighting on structural brain networks in the UK biobank. NeuroImage 2020; 211: 116443. 31927129 10.1016/j.neuroimage.2019.116443 PMC7085460

[51] Jenkinson M Bannister P Brady M , et al. Improved optimization for the robust and accurate linear registration and motion correction of brain images. NeuroImage 2002; 17: 825–841. 12377157 10.1016/s1053-8119(02)91132-8

[52] Shirer WR Jiang H Price CM , et al. Optimization of rs-fMRI pre-processing for enhanced signal-noise separation, test-retest reliability, and group discrimination. NeuroImage 2015; 117: 67–79. 25987368 10.1016/j.neuroimage.2015.05.015

[53] Kalpouzos G Chételat G Baron JC , et al. Voxel-based mapping of brain gray matter volume and glucose metabolism profiles in normal aging. Neurobiol Aging 2009; 30: 112–124. 17630048 10.1016/j.neurobiolaging.2007.05.019

[54] Wu K Taki Y Sato K , et al. Age-related changes in topological organization of structural brain networks in healthy individuals. Hum Brain Mapp 2012; 33: 552–568. 21391279 10.1002/hbm.21232 PMC6870030

[55] Tong Y Hocke LM Frederick BB. Low frequency systemic hemodynamic “noise” in resting state BOLD fMRI: characteristics, causes, implications, mitigation strategies, and applications. Front Neurosci 2019; 13: 787. 31474815 10.3389/fnins.2019.00787 PMC6702789

[56] Geerligs L Cam CAN Henson RN. Functional connectivity and structural covariance between regions of interest can be measured more accurately using multivariate distance correlation. NeuroImage 2016; 135: 16–31. 27114055 10.1016/j.neuroimage.2016.04.047 PMC4922835

[57] Huang SY Hsu JL Lin KJ , et al. Characteristic patterns of inter- and intra-hemispheric metabolic connectivity in patients with stable and progressive mild cognitive impairment and Alzheimer’s disease. Sci Rep 2018; 8: 13807. 30218083 10.1038/s41598-018-31794-8 PMC6138637

[58] Vanicek T Hahn A Traub-Weidinger T , et al. Insights into intrinsic brain networks based on graph theory and PET in right- compared to left-sided temporal lobe epilepsy. Sci Rep 2016; 6: 28513. 27349503 10.1038/srep28513 PMC4923886

[59] Hagmann P Cammoun L Gigandet X , et al. Mapping the structural core of human cerebral cortex (Friston KJ, ed.). PLoS Biol 2008; 6: e159. 18597554 10.1371/journal.pbio.0060159 PMC2443193

[60] Savio A Fünger S Tahmasian M , et al. Resting-state networks as simultaneously measured with functional MRI and PET. J Nucl Med 2017; 58: 1314–1317. 28254868 10.2967/jnumed.116.185835 PMC6944183

[61] Sala A Lizarraga A Ripp I , et al. Static versus functional PET: making sense of metabolic connectivity. Cereb Cortex 2022; 32: 1125–1129. 34411237 10.1093/cercor/bhab271

[62] Wang J Khosrowabadi R Ng KK , et al. Alterations in brain network topology and structural-functional connectome coupling relate to cognitive impairment. Front Aging Neurosci 2018; 10: 404. 30618711 10.3389/fnagi.2018.00404 PMC6300727

[63] Mechelli A Friston KJ Frackowiak RS , et al. Structural covariance in the human cortex. J Neurosci 2005; 25: 8303–8310. 16148238 10.1523/JNEUROSCI.0357-05.2005 PMC6725541

[64] Draganski B May A. Training-induced structural changes in the adult human brain. Behav Brain Res 2008; 192: 137–142. 18378330 10.1016/j.bbr.2008.02.015

[65] Zhao K Zheng Q Che T , et al. Regional radiomics similarity networks (R2SNs) in the human brain: reproducibility, small-world properties and a biological basis. Network Neuroscience 2021; 5: 783–797. 34746627 10.1162/netn_a_00200 PMC8567836

[66] Betzel RF Griffa A Hagmann P , et al. Distance-dependent consensus thresholds for generating group-representative structural brain networks. Netw Neurosci 2019; 3: 475–496. 30984903 10.1162/netn_a_00075 PMC6444521

[67] Donahue CJ Sotiropoulos SN Jbabdi S , et al. Using diffusion tractography to predict cortical connection strength and distance: a quantitative comparison with tracers in the monkey. J Neurosci 2016; 36: 6758–6770. 27335406 10.1523/JNEUROSCI.0493-16.2016 PMC4916250

[68] Calamante F. The seven deadly sins of measuring brain structural connectivity using diffusion MRI streamlines fibre-tracking. Diagnostics 2019; 69: 115. 10.3390/diagnostics9030115 PMC6787694 31500098

