# Supplementary material for: Similarity between structural and proxy estimates of brain connectivity
Source: J Cereb Blood Flow Metab. 2023 Sep 29;44(2):284–95. doi: 10.1177/0271678X231204769 (PMC10993877; doi:10.1177/0271678X231204769)
Supplement: sj-pdf-1-jcb-10.1177_0271678X231204769 - Supplemental material for Similarity between structural and proxy estimates of brain connectivity [file sj-pdf-1-jcb-10.1177_0271678X231204769.pdf]

Supplementary Material

Article: Similarity between structural and proxy estimates of brain connectivity

Authors: Aldana Lizarraga, Isabelle Ripp, Arianna Sala, Kuangyu Shi, Marco Düring, Kathrin Koch, Igor Yakushev

Corresponding author: Igor Yakushev, MD. Dept. of Nuclear Medicine, Technical University of Munich, Ismaninger Str. 22, 81675 Munich, Germany. E-mail: igor.yakushev@tum.de

Calculation of convergence ratio by chance

Given a random network X of connection density δ, defined as the proportion on non-zero elements in its adjacency matrix, the probability of any i,j element being non-zero is P(xij ≠ 0) = δ. Therefore, the probability of any element being non-zero in two networks X, Y of identical density δ is

$$P(x_{ij} \neq 0 \wedge y_{ij} \neq 0) = P(x_{ij} \neq 0) \cdot P(y_{ij} \neq 0) = \delta \cdot \delta$$

If N is the total number of connections in the networks, the expected number of connections in each network is Nδ; while the expected number of common connections, or connections in the intersection of the two networks is

$$N \cdot P(x_{ij} \neq 0 \wedge y_{ij} \neq 0) = N \cdot \delta^2$$

Then, the CR (number of common connections divided by the average of the connections in the two networks, expressed as a percentage) expected by chance between two random networks is

$$CR_{random} = \frac{N \cdot \delta^2}{\frac{N\delta + N\delta}{2}} \cdot 100\% = \delta \cdot 100\%$$

Adaptation of the AAL2 atlas

To minimize partial volume effects in the PET data, we adapted the 120-region AAL2 atlas by reducing the number of small regions. To this end, we calculated volumes of the regions in the individual T1 space after intersection with the corresponding 0.5 grey matter mask. Then, we computed mean and standard deviation of values across subjects. Included were all regions with a mean volume above 512 mm^3, i.e. 2 x FWHM of spatial resolution of our PET/MR system (~4.0 mm) in all directions. As a result, we obtained a 106-region parcellation, see Supplementary Table 1.

Supplementary Figures

Figure S1. SC connectivity matrix (left) and SC connection weight distribution (right) whithout threshold

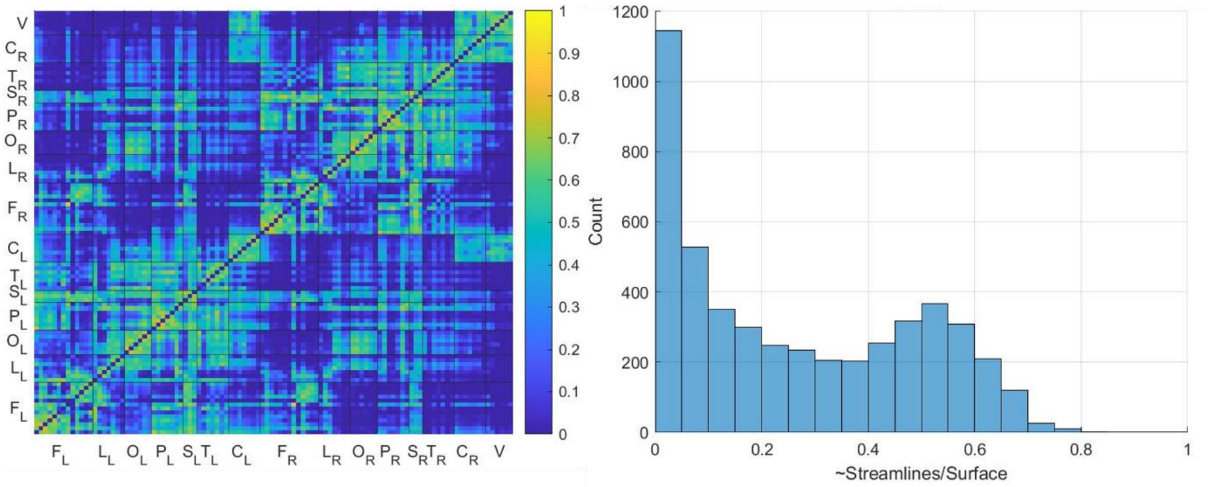

Figure S2. Distribution of raw (white), structurally consistent (blue) and 34% strongest (transparent red) connections for FC (left), FDGcov (middle) and GMVcov (right)

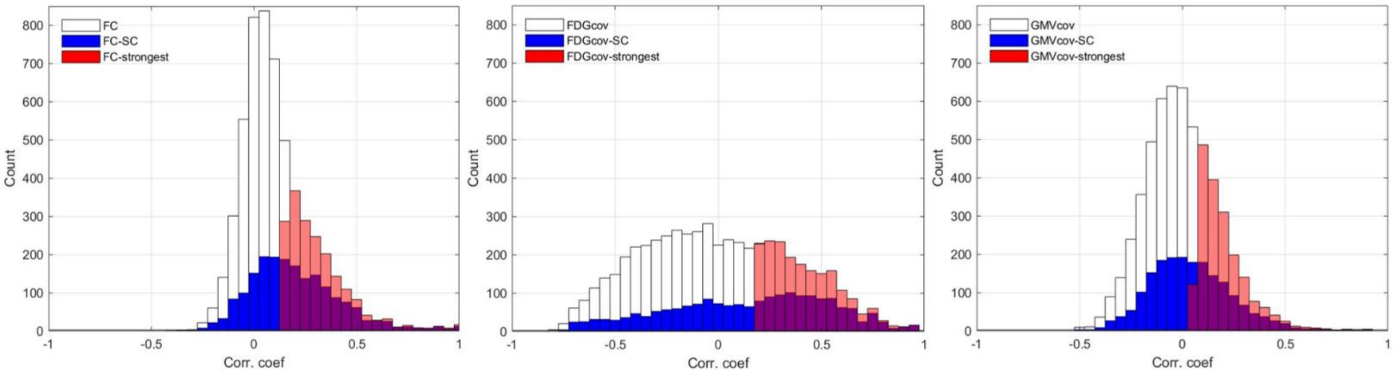

Figure S3. Scatter plots of positive connection weights and Spearman correlation coefficients (SCC) between SC and proxy estimates

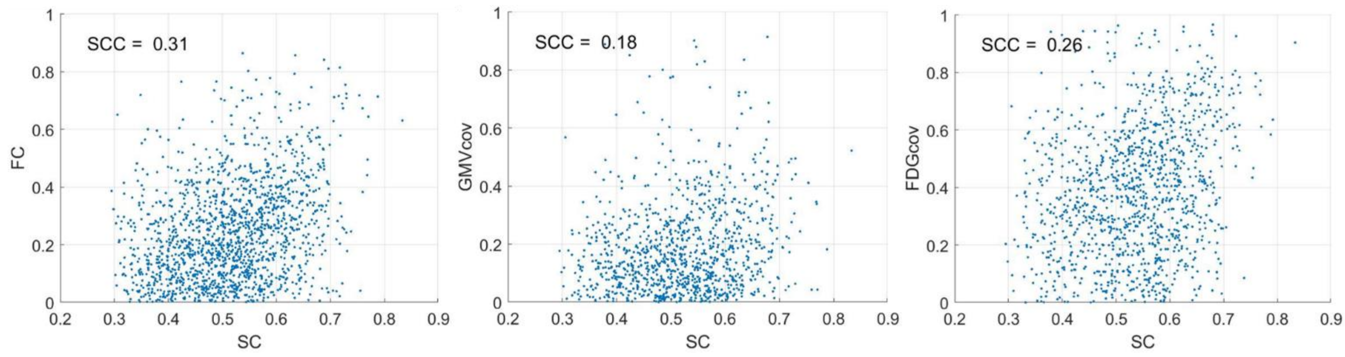

Figure S4. Scatter plots of connection weights and Spearman correlation coefficients (SCC) between 3 proxy estimates; all possible connections (5565) were considered.

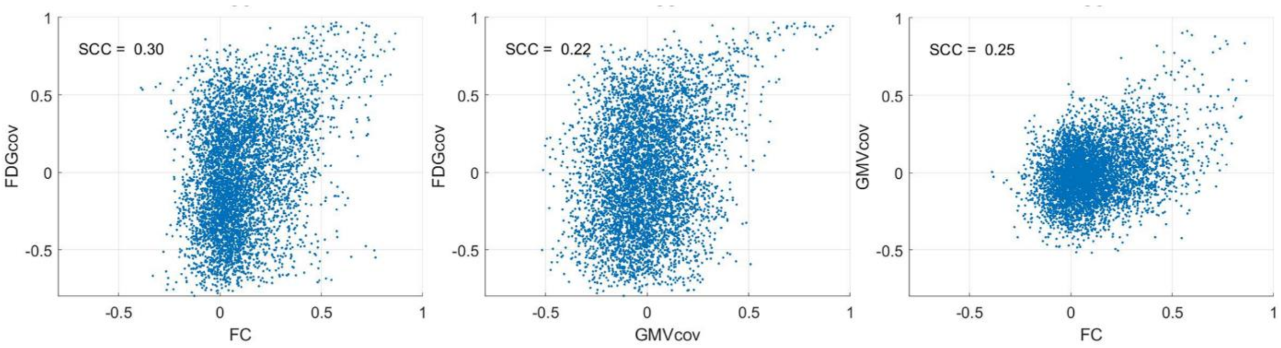

Figure S5. Convergence ratio (CR) between the networks as function of sparsity level; the percentages indicate average CRs over the sparsity range

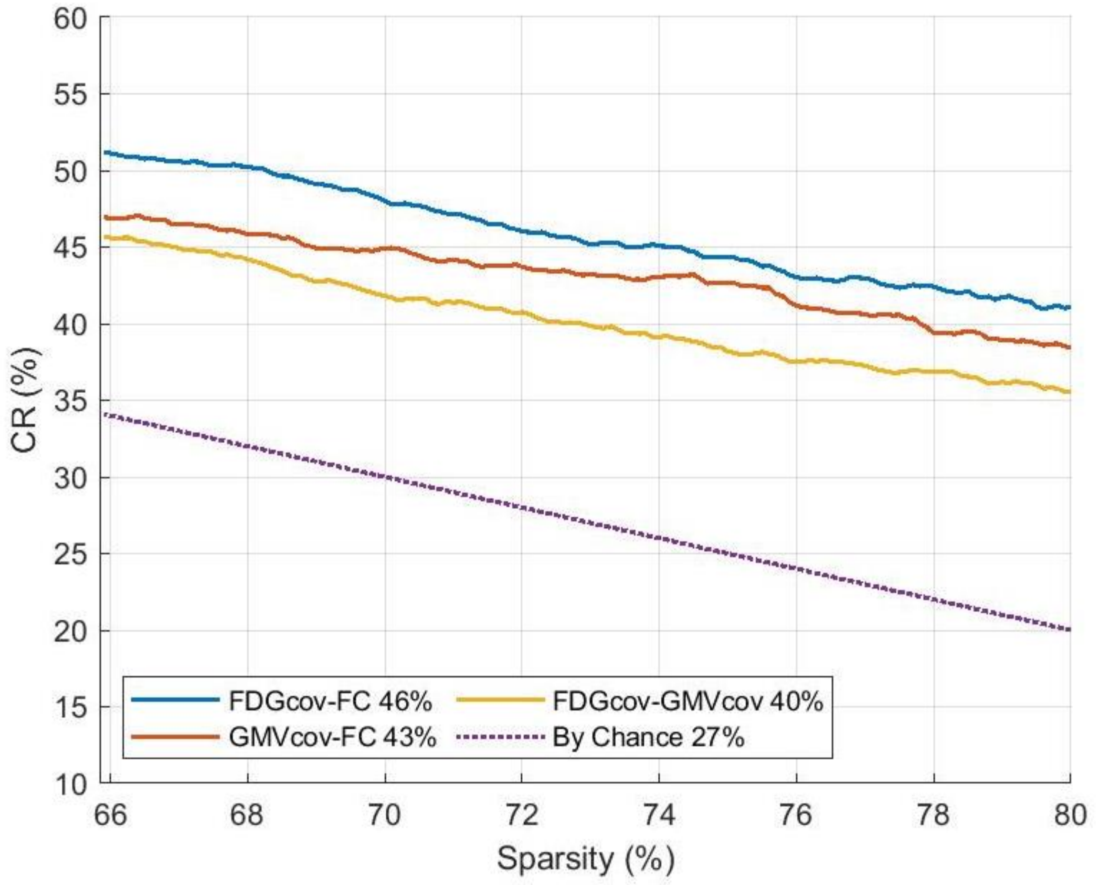

Supplementary Tables

Table S1. List of the 106 regions adapted from the AAL2 atlas

Region	In AAL2	Lobe	Region	In AAL2	Lobe
1-Precentral-L	1	Frontal	54-Postcentral-R	62	Parietal
2-Precentral-R	2	Frontal	55-Parietal-Sup-L	63	Parietal
3-Frontal-Sup-2-L	3	Frontal	56-Parietal-Sup-R	64	Parietal
4-Frontal-Sup-2-R	4	Frontal	57-Parietal-Inf-L	65	Parietal
5-Frontal-Mid-2-L	5	Frontal	58-Parietal-Inf-R	66	Parietal
6-Frontal-Mid-2-R	6	Frontal	59-SupraMarginal-L	67	Parietal
7-Frontal-Inf-Opera-L	7	Frontal	60-SupraMarginal-R	68	Parietal
8-Frontal-Inf-Oper-R	8	Frontal	61-Angular-L	69	Parietal
9-Frontal-Inf-Tri-L	9	Frontal	62-Angular-R	70	Parietal
10-Frontal-Inf-Tri-R	10	Frontal	63-Precuneus-L	71	Parietal
11-Frontal-Inf-Orb-2-L	11	Frontal	64-Precuneus-R	72	Parietal
12-Frontal-Inf-Orb-2-R	12	Frontal	65-Paracentral-Lobule-L	73	Parietal
13-Rolandic-Oper-L	13	Frontal	66-Paracentral-Lobule-R	74	Parietal
14-Rolandic-Oper-R	14	Frontal	67-Caudate-L	75	Subcortical
15-Supp-Motor-Area-L	15	Frontal	68-Caudate-R	76	Subcortical
16-Supp-Motor-Area-R	16	Frontal	69-Putamen-L	77	Subcortical
17-Olfactory-L	17	Frontal	70-Putamen-R	78	Subcortical
18-Olfactory-R	18	Frontal	71-Thalamus-L	81	Subcortical
19-Frontal-Sup-Medial-L	19	Frontal	72-Thalamus-R	82	Subcortical
20-Frontal-Sup-Medial-R	20	Frontal	73-Fusiform-L	59	Temporal
21-Frontal-Med-Orb-L	21	Frontal	74-Fusiform-R	60	Temporal
22-Frontal-Med-Orb-R	22	Frontal	75-Heschl-L	83	Temporal
23-Rectus-L	23	Frontal	76-Heschl-R	84	Temporal
24-Rectus-R	24	Frontal	77-Temporal-Sup-L	85	Temporal
25-OFCmed-ant-post-lat-L	25+27+29+31	Frontal	78-Temporal-Sup-R	86	Temporal
26-OFCmed-ant-post-lat-R	26+28+30+32	Frontal	79-Temporal-Pole-Sup-L	87	Temporal
27-Insula-L	33	Limbic	80-Temporal-Pole-Sup-R	88	Temporal
28-Insula-R	34	Limbic	81-Temporal-Mid-L	89	Temporal
29-Cingulate-Ant-L	35	Limbic	82-Temporal-Mid-R	90	Temporal
30-Cingulate-Ant-R	36	Limbic	83-Temporal-Pole-Mid-L	91	Temporal
31-Cingulate-Mid-L	37	Limbic	84-Temporal-Pole-Mid-R	92	Temporal
32-Cingulate-Mid-R	38	Limbic	85-Temporal-Inf-L	93	Temporal

33-Cingulate-Post-L	39	Limbic	86-Temporal-Inf-R	94	Temporal
34-Cingulate-Post-R	40	Limbic	87-Cerebellum-Crus1-L	95	Posterior Fossa
35-Hippocampus-L	41	Limbic	88-Cerebellum-Crus1-R	96	Posterior Fossa
36-Hippocampus-R	42	Limbic	89-Cerebellum-Crus2-L	97	Posterior Fossa
37-ParaHippocampal-L	43	Limbic	90-Cerebellum-Crus2-R	98	Posterior Fossa
38-ParaHippocampal-R	44	Limbic	91-Cerebellum-3-4-5-L	99+101	Posterior Fossa
39-Amygdala-L	45	Limbic	92-Cerebellum-3-4-5-R	100+102	Posterior Fossa
40-Amygdala-R	46	Limbic	93-Cerebellum-6-L	103	Posterior Fossa
41-Calcarine-L	47	Occipital	94-Cerebellum-6-R	104	Posterior Fossa
42-Calcarine-R	48	Occipital	95-Cerebellum-7b-L	105	Posterior Fossa
43-Cuneus-L	49	Occipital	96-Cerebellum-7b-R	106	Posterior Fossa
44-Cuneus-R	50	Occipital	97-Cerebellum-8-10-L	107+111	Posterior Fossa
45-Lingual-L	51	Occipital	98-Cerebellum-8-10-R	108+112	Posterior Fossa
46-Lingual-R	52	Occipital	99-Cerebellum-9-L	109	Posterior Fossa
47-Occipital-Sup-L	53	Occipital	100-Cerebellum-9-R	110	Posterior Fossa
48-Occipital-Sup-R	54	Occipital	101-Vermis-1-2-3	113+114	Posterior Fossa
49-Occipital-Mid-L	55	Occipital	102-Vermis-4-5	115	Posterior Fossa
50-Occipital-Mid-R	56	Occipital	103-Vermis-6	116	Posterior Fossa
51-Occipital-Inf-L	57	Occipital	104-Vermis-7	117	Posterior Fossa
52-Occipital-Inf-R	58	Occipital	105-Vermis-8	118	Posterior Fossa
53-Postcentral-L	61	Parietal	106-Vermis-9-10	119+120	Posterior Fossa

Only the regions 79-Pallidum-L and 80-Pallidum-R from the AAL2 atlas were removed for this study.

Table S2. Robustness analyses for proxy estimates of connectivity

	SCC						
	O	a)	b)	c)	d)	e)	f)

FDG_{cov}-FC	0.30	0.27	0.30*	0.30*	0.30*	0.20	0.29
FDG_{cov}-GMV_{cov}	0.22	0.21	0.22*	0.22*	0.22*	0.14	0.17
GMV_{cov}-FC	0.25	0.23	0.25*	0.25*	0.25*	0.19	0.20
				CR			
FDG_{cov}-FC	46	44	46	63	50	40	45
FDG_{cov}-GMV_{cov}	40	39	40	60	44	37	38
GMV_{cov}-FC	43	40	43	61	46	38	42
CR by chance	27	27	27	53	32	27	30
Min sparsity	65.8	66.1	65.8	13.3	55.0	65.8	63.8
Max sparsity	80.0	79.8	80.0	80.0	80.0	80.0	77.4

Similarity metrics obtained under modified conditions relative to the original (O) ones: a) Test-retest reproducibility, b) Omitting the Gaussian resampling step of SC, c) Omitting a proportional threshold to SC matrix, d) Applying a 50% proportional threshold to the SC matrix, e) Regressing out distance from all connectivity estimates, f) limiting the analysis to the cerebral cortex. SCC: Spearman correlation coefficient. CR: mean convergence ratio computed over the corresponding sparsity range [Min sparsity, Max sparsity]. *: results are the same as the original ones because the similarity is not affected by the SC network.
